# JIA affected sibling pairs present high correlation for ANA and ILAR category

**DOI:** 10.1186/1546-0096-9-S1-P193

**Published:** 2011-09-14

**Authors:** G Filocamo, C Malattia, I Foeldvari, V Stanevicha, S Nielsen, T Herlin, C Pruunsild, F Zulian, Z Balogh, F Dressler, I Rumba, MG Alpigiani, E Cortis, F Falcini, R Trauzeddel, G Calcagno, L Lepore, M Alessio, DN Glass, SD Thompson, A Martini, N Ruperto

**Affiliations:** 1 IRCCS G. Gaslini, Pediatria II, PRINTO, Italy

## Aims

1)To investigate the clinical phenotypes and demographic characteristics of affected sibling pairs (ASPs) with juvenile idiopathic arthritis (JIA)

2)To provide an international resource of JIA DNA samples and a base of knowledge from which all genes contributing to the pathogenesis of JIA can be identified.

## Methods

This is a cross-sectional multicentric study in which all paediatric rheumatology centres belonging to the PRINTO network were asked to participate. PRINTO asked to provide demographic and clinical characteristics of the ASPs through electronic format and to collect DNA samples of JIA familiar cases, including all first degree relatives, deriving from the different centres.

## Results

Table 1. Demographic, clinical features and concordance between the sibs (2 or more within the same family) of the 106 individual affected sibpairs with JIA

**Table 1:** 

	N (%)	Mean (SD)	Median	Min	Max	Conc.
Male Female	25 (24) 81 (76)					0.24 0.79
Onset age –years		6.6 (4.4)	6.2	0.5	16.3	

JIA category Systemic arthritis Oligoarthritis persistent Oligoarthritis extended Polyarthritis RF negative Polyarthritis RF positive Psoriatic arthritis Enthesitis related arthritis Other	0 (0) 61 (57.6) 18 (17) 21 (19.8) 2 (1.9) 3 (2.8) 0 (0) 1 (0.9)					0.79 0.85 0.5 0.9 1 0.67 0

Presence of ANA Absence of ANA	56 (56) 44 (44)					0.78 0.68
Presence of iritis Absence of iritis	11 (10) 86 (81)					0.36 0.9

## Conclusion

Preliminary results confirm the findings of earlier studies showing familial aggregation of clinical features among ASPs. In our study we observed high concordance of the presence of antinuclear antibodies (ANA), providing evidence for a genetic background in this disease. The DNA samples collected will allow to develop future studies on JIA.

